# Comparison of different probiotics in the treatment of acute diarrhea in children: A protocol for systematic review and network meta-analysis

**DOI:** 10.1097/MD.0000000000028899

**Published:** 2022-03-18

**Authors:** Xiao Feng, LiJuan Zhuang, Ling Chen, Hongying Zhao, Rui Huang, ZhiFeng Guo

**Affiliations:** *Zhongshan Hospital, Xiamen University, Xiamen, Fujian, China.*

**Keywords:** acute diarrhea, children, network meta-analysis, probiotics, protocol

## Abstract

**Background:**

Acute diarrhea has a serious impact on the health and lives of children. Previous studies have shown that probiotics have positive and reliable efficacy in the treatment of acute diarrhea in children, but the efficacy of different types of probiotics varies. This study will evaluate the clinical efficacy of different kinds of probiotics in the treatment of acute diarrhea in children by means of network meta-analysis.

**Methods:**

According to the retrieval strategy, randomized controlled studies on probiotics in the treatment of acute diarrhea in children will be searched from PubMed, Embase, Web of Science, the Cochrane Library, CNQI, Wanfang, VIP, and Chinese biomedical databases. The retrieval time limit will be from the establishment of the database to January 2022. The quality level of the included studies will be assessed using the Cochrane Risk Bias Assessment Tool and the strength of evidence for outcome measures will be assessed using the Grading of Recommendation Assessment, Development, and Evaluation method. All data analysis will be performed by Revman5.3, Gemtc 0.14.3 and Stata 14.0.

**Results:**

This study will evaluate the efficacy of different kinds of probiotics in the treatment of acute diarrhea in children by evaluating diarrhea duration, stool frequency, length of hospital stay, adverse reactions, etc.

**Conclusions:**

This study will provide a reliable evidence-based basis for the selection of probiotics for the treatment of acute diarrhea in children.

**Ethics and dissemination:**

Private information from individuals will not be published. This systematic review also does not involve endangering participant rights. Ethical approval will not be required. The results may be published in a peer-reviewed journal or disseminated at relevant conferences.

**OSF registration number:**

DOI 10.17605/OSF.IO/MNJAE.

## 1. Introduction

The World Health Organization (WHO) defines acute diarrhea as the discharge of loose stools or liquid stools ≥ 3 times per day for ≥ 3days and < 14days.^[1]^ According to statistics, every child has about 2∼3 times of diarrhea within a year,^[2]^ whereas in developing countries, every child has 6∼12 times of diarrhea per year.^[3]^ Acute diarrhea has become the second leading cause of death in children under 5 years old,^[4]^ causing about 525,000 deaths of children under 5 years old every year, most of which occur in developing countries.^[5]^ Other direct effects of acute diarrhea include growth disorders, malnutrition and impaired cognitive development in children.^[6]^

At present, the treatment for acute diarrhea in children mainly consists of water and electrolytes supplementation, and adjuvant anti-infective therapy when pathogens test positive.^[7,8]^ Due to limited treatment methods and relatively high incidence, current population control measures for childhood diarrhea mainly focus on prevention strategies, including calling for breastfeeding, strengthening nutrition, improving drinking water sanitation and personal hygiene, and supplementing vitamins.^[8,9]^ Oral probiotics is another clinically proven positive treatment for acute diarrhea in children,^[5]^ which has been applied to respiratory system, digestive system, skin and other fields and has received positive feedback.^[10,11]^

Probiotics is a general term for a group of active microorganisms that have a certain amount in human body and can promote the health of the host.^[12]^ Through colonization in the intestine, probiotics can effectively improve the intestinal microenvironment, reduce intestinal pH, prevent intestinal bacterial translocation, and prevent a variety of intestinal diseases.^[13]^ Modern studies have shown that probiotics not only have immunomodulatory effects, but also can regulate intestinal flora, maintain intestinal microecological balance, and enhance the barrier function of intestinal epithelium.^[13,14]^ Current evidence suggests that multiple types of probiotics have a positive effect on acute diarrhea in children. However, different types of probiotics have different clinical effects. For example, *Lactobacillus rhamnosus* and *Saccharomyces boulardii* can significantly shorten the duration of diarrhea and hospital stay in children with acute diarrhea compared with placebo.^[15,16]^ Compared with placebo, *Lactobacillus reuteri* showed no difference in shortening the duration of diarrhea but shortened the length of hospital stay.^[17]^

In clinical practice, doctors often have to select the best treatment among multiple interventions. Traditional metaanalysis can only compare the advantages and disadvantages of 2 interventions in disease treatment, but cannot compare the efficacy differences of multiple interventions at the same time. Network meta-analysis combines evidence from direct and indirect comparisons to simultaneously evaluate all interventions in the same body of evidence and rank efficacy. To investigate the efficacy differences of different types of probiotics in the treatment of acute diarrhea in children, we conducted this network meta-analysis to provide comprehensive evidence for the selection of optimal probiotics for the clinical treatment of acute diarrhea in children.

## 2. Methods

### 2.1. Protocol register

This research scheme was conducted under the guidance of preferred reporting items for systematic reviews and metaanalyses protocols. This program was registered on the Open Science Framework in January 18, 2022. (Registration number: DOI 10.17605/OSF.IO/MNJAE).

### 2.2. Ethics

Since this program does not require patient recruitment and collection of personal information, it does not require the approval of ethics committee.

### 2.3. Eligibility criteria

- Types of study: randomized controlled trial, the language will be limited to Chinese literature and English.
- Subjects of study: children diagnosed with acute diarrhea (age < 16years, diarrhea duration < 14days).
- Intervention measures: the treatment group will be given a probiotic regimen alone (such as *L rhamnosus*, *S boulardii*, *Bacillus clausii*, *Bifidobacterium lactate*, *Lactobacillus acidophilus*, etc), and the control group will be given placebo, drugs or a separate probiotic regimen different from the treatment group.
- Exclusion criteria:
  - Studies whose intervention measures were a mixture of probiotics or probiotics combined with antibiotics, etc.
  - Studies which is unable to extract relevant data from published results, and unable to obtain original data after contacting the author;
  - Studies published repeatedly;
  - Studies whose literatures are abstract, animal research, and cadaver research.

### 2.4. Outcome indicator

- Primary outcome indicator: duration of diarrhea, number of stools;
- Secondary outcome indicator: length of hospital stay, adverse reactions.

### 2.5. Search strategy

The 2 researchers will search independently through search databases, including CNKI, Wanfang Data Knowledge Service Platform, VIP Information Chinese Journal Service Platform (VIP), and China Biomedical Database. The retrieval time will be from the establishment of the database to January 2022. Chinese keywords: “acute diarrhea” (ji xing fu xie/ji xing xie xie), “children” (er tong), “probiotics” (yi sheng jun), “*Lactobacillus rhamnosus*” (shu li tang ru gan jun), “*Saccharomyces boulardii*” (bu la shi jiao mu jun), “*Bifidobacterium lactate*” (ru suan shuang qi gan jun), “*Bacillus clausii*” (ke lao shi ya bao gan jun), “colibacillus” (da chang gan jun); English keywords: “acute diarrhea”, “probiotic”, “lactobacillus”, “saccharomyces”, “bacillus”, etc. The 2 researchers will select the included literature independently according to the inclusion and exclusion criteria. In case of any disagreement, the decision will be made after consultation with the third researcher. PubMed retrieval strategies are shown in Figure 1.

**Figure:**
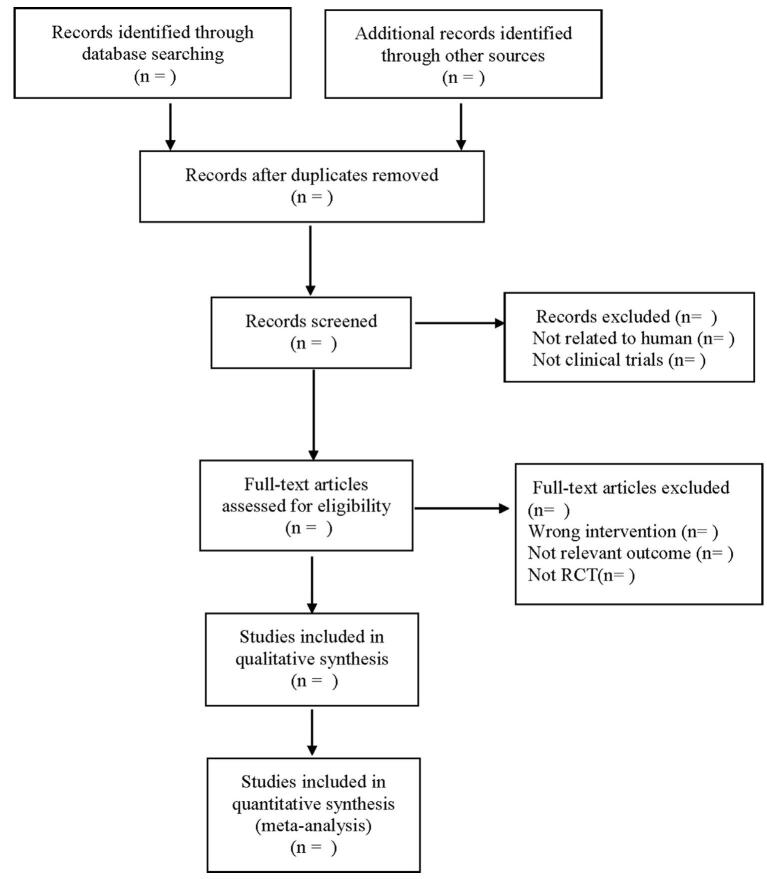
**Figure 1.** Flow diagram.

### 2.6. Data screening and extraction

The results will be imported into Endnote, and complete the preliminary screening by reading the titles and abstracts of all literature retrieved. After the preliminary screening, the full text of the literature will be obtained, the inclusion or exclusion of the literature records will be determined by reading the full text, and the excluded literature record reasons for exclusion. Excel forms will be developed in advance to collect the first author, publication year, age of patients, number of patients, intervention measures, etc. The literature screening process is shown in Table 1.

**Table 1:** Retrieval strategy of PubMed.

**Number**	**Search terms**
#1	Probiotics [MeSH]
#2	Lactobacillus [Title/Abstract]
#3	Saccharomyces [Title/Abstract]
#4	Bifidobacterium [Title/Abstract]
#5	Boulardii [Title/Abstract]
#6	Bacillus [Title/Abstract]
#7	Subtilis [Title/Abstract]
#8	Enterococcus faecium [Title/Abstract]
#9	Rhamnosus [Title/Abstract]
#10	Thermophilus [Title/Abstract]
#11	Acidophilus [Title/Abstract]
#12	Plantarum [Title/Abstract]
#13	Bulgaricus [Title/Abstract]
#14	Bifidum [Title/Abstract]
#15	#1 OR #2 OR #3 OR #4 OR #5 OR #6 OR #7 OR #8 OR #9 OR #10 OR #11 OR #12 OR #13 OR #14
#16	Diarrhea [MeSH]
#17	Diarrheas [Title/Abstract]
#18	Diarrhoea [Title/Abstract]
#19	Diarrh [Title/Abstract]
#20	#16 OR #17 OR #18 OR #19
#21	Children [Title/Abstract]
#22	Child [Title/Abstract]
#23	Pediatric [Title/Abstract]
#24	Pediatr [Title/Abstract]
#25	#21 OR #22 OR #23 OR #24
#26	#15 AND #20 AND #25

### 2.7. Literature quality assessment

Two investigators will independently evaluate the quality of the included studies using the Cochrane Risk Bias Assessment Tool, and a third investigator will participate in the discussion when there is disagreement. The evaluation indicators will include random sequence generation, allocation hiding, blind method, integrity of outcome data, selective reporting of research results and other sources of bias. According to these indicators, the included literatures will be evaluated as “high risk of bias”, “low risk of bias”, and “unknown”.

### 2.8. Statistical analysis

Stata14.0 software will be used to draw an evidence network diagram to present the comparative relationship between interventions of each outcome indicator. Network meta-analysis will be conducted using GeMTC14.3 based on Bayesian framework. The effect value of binary variable will be represented by odd ratio, and the effect value of continuous variables will be represented by mean different. The 95% confidence interval will be used to represent the statistical analysis results. Use Markov Chain Monte Carlo fitting consistent model for Bayesian inference, use 4 chains for simulation, set the number of iterations as 50,000 times (the first 20,000 times for annealing, the last 30,000 times for sampling). Potential scale reduction factor will be used to reflect the convergence degree of the model. When potential scale reduction factor is close to or equal to 1, it indicates that the data convergence is good and the obtained results are highly reliable.

### 2.9. Assessment of inconsistency

When there is a closed ring between each intervention, inconsistency test will be required. Stata14.0 will be used for Z test to evaluate the consistency between direct and indirect comparison results. If *P* ≥ .05, it means that direct comparison and indirect comparison are less likely to be inconsistent; if *P* < .05, there is a greater possibility of inconsistency between direct comparison and indirect comparison, and fitting inconsistency analysis is required. Stata14.0 will be used to calculate the surface under the cumulative ranking curves of different interventions, and the larger the surface under the cumulative ranking curves value, the better the therapeutic effect of the intervention. Finally, a comparative-correction map will be drawn to assess whether the small sample effect exists.

### 2.10. Sensitivity analysis

To verify the robustness of the results, sensitivity analysis will be performed on the results of each outcome indicator.

### 2.11. Assessment of publication bias

If the number of studies is sufficient (n ≥ 10), we will evaluate the publication bias of the included studies using funnel plots^[18]^ to detect the existence of publication bias or small sample effect.

### 2.12. Evidence quality evaluation

We will use the Grading of Recommendation Assessment, Development, and Evaluation scoring method to grade the evidence of the outcome index.^[19]^ The evaluation content includes bias risk, indirectness, inconsistency, inaccuracy, and publication bias. And the quality of evidence will be rated as high, medium, low or very low.

## 3. Discussion

There are more than 1000 species of bacteria inhabiting human intestine,^[20]^ most of which are colonized in the human colon. The intestinal microecosystem is the largest and most important microecosystem of the body, which plays an important role in the health and nutrition of the host and is a key factor in activating and maintaining the physiological functions of the intestine.^[21]^ Some studies have pointed out that the type and number of probiotics in human intestinal tract can reflect the health status of human body.^[22]^

When children are affected by age, environment, diet, medication (such as antibiotics) and other factors, intestinal microecological imbalance, namely imbalance of intestinal flora can be caused, and then result in the occurrence of diarrhea and other diseases and related symptoms.^[23]^ A large number of clinical studies have confirmed that certain kinds of probiotics can shorten the duration of diarrhea and hospital stay in children with acute diarrhea.^[5,8]^ Traditional meta-analysis can evaluate the efficacy of single probiotics or the overall efficacy of probiotics, but the efficacy of probiotics is strain specific, and different probiotics also have different efficacy.^[9]^ Traditional meta-analysis cannot meet the needs of selecting the best from many interventions in clinical practice. Network meta-analysis is an extension of traditional meta-analysis, which can compare multiple interventions at the same time. Indirect comparison can also be made when there is no direct comparison of interventions, and the best intervention can be obtained based on evidence.^[24]^ In order to explore the efficacy differences of different probiotics in the treatment of acute diarrhea in children and provide reference evidence for clinical application, this study will use the method of network meta-analysis to explore the efficacy differences of commonly used probiotics at present in clinical practice, so as to provide evidence-based basis for clinical decision makers to select the optimal scheme.

However, there are some limitations in our study: due to the limitation of language retrieval, we will only include Chinese and English literatures, which may cause selection bias; differences in age, severity of disease, treatment duration and other factors will increase the possibility of heterogeneity. Nevertheless, we believe that the results of this study will help identify the best probiotic regimen for treating acute diarrhea in children.

## Author contributions

**Data collection:** Xiao Feng and LiJuan Zhuang.

**Funding support:** Hongying Zhao.

**Resources:** LiJuan Zhuang and Ling Chen.

**Software operating:** Ling Chen and ZhiFeng Guo.

**Supervision:** Xiao Feng and Rui Huang.

**Writing** - **original draft:** Xiao Feng and LiJuan Zhuang.

**Writing** - **review & editing:** Xiao Feng and Hongying Zhao.
